# Hair-bundle proteomes of avian and mammalian inner-ear utricles

**DOI:** 10.1038/sdata.2015.74

**Published:** 2015-12-08

**Authors:** Phillip A. Wilmarth, Jocelyn F. Krey, Jung-Bum Shin, Dongseok Choi, Larry L. David, Peter G. Barr-Gillespie

**Affiliations:** 1 Department of Biochemistry and Molecular Biology, Oregon Health and Science University, Portland, OR, USA; 2 Oregon Hearing Research Center & Vollum Institute, Oregon Health & Science University, Portland, OR, USA; 3 Department of Neuroscience, University of Virginia, Charlottesville, VA, USA; 4 Department of Public Health & Preventive Medicine, Oregon Health & Science University, Portland, OR, USA

**Keywords:** Hair cell, Proteomic analysis, Sensory processing

## Abstract

Examination of multiple proteomics datasets within or between species increases the reliability of protein identification. We report here proteomes of inner-ear hair bundles from three species (chick, mouse, and rat), which were collected on LTQ or LTQ Velos ion-trap mass spectrometers; the constituent proteins were quantified using MS2 intensities, which are the summed intensities of all peptide fragmentation spectra matched to a protein. The data are available via ProteomeXchange with identifiers PXD002410 (chick LTQ), PXD002414 (chick Velos), PXD002415 (mouse Velos), and PXD002416 (rat LTQ). The two chick bundle datasets compared favourably to a third, already-described chick bundle dataset, which was quantified using MS1 peak intensities, the summed intensities of peptides identified by high-resolution mass spectrometry (PXD000104; updated analysis in PXD002445). The mouse bundle dataset described here was comparable to a different mouse bundle dataset quantified using MS1 intensities (PXD002167). These six datasets will be useful for identifying the core proteome of vestibular hair bundles.

## Background & Summary

The inner ear’s sensory hair cells detect mechanical stimuli from the environment using their hair bundles, each of which consists of ~100 actin-filled stereocilia and an axonemal kinocilium^1^. Bundle deflection modulates the open probability of cation-selective transduction channels; channel opening produces receptor potentials, and the ensuing excitation propagates to the central nervous system. Understanding the complete protein composition of the bundle is essential for describing the process of mechanotransduction^2^; the bundle’s structure specifies the sensitivity of the system to sound, as well as its ability to adapt to prolonged stimuli.

A wide variety of methods for quantitation of proteins using mass spectrometry are available^3,4^. While targeted proteomics offers the greatest accuracy and precision^5^, these methods require prior knowledge of the identities of quantified proteins. By contrast, shotgun proteomics^6,7^, which is used as a discovery technique, offers quantitation even of unexpected proteins. While numerous quantitation methods have been developed for shotgun experiments, label-free detection using spectral counts or ion-current intensities are the most easily implemented^3,4^. Measurement of ion-current intensity can be conducted at the MS1 (peptide) or MS2 (fragmentation products) stages. By comparing results with standard proteins diluted in an *E. coli* protein background, we demonstrated that normalized summed MS2 intensities derived from ion-trap mass spectrometer data yields quantitation results that are nearly as accurate as measurement of those same proteins with MS1 peak areas using an Orbitrap mass spectrometer^8^. This observation suggests that workhorse ion-trap instruments can be used for estimation of the concentrations of abundant and moderately abundant proteins in a complex mixture like hair bundles.

When carrying out initial surveys of a complex protein sample, such as isolated hair bundles, shotgun mass spectrometry in principle allows for detection of all proteins in a mixture. Unfortunately, when combined with the enormous concentration range for proteins in cellular extracts, the limited dynamic range of mass spectrometers—even in fast modern instruments—ensures the sampling of many proteins will be stochastic^7^. Proteins are observed inconsistently from sample to sample, and protein abundances are accordingly inaccurately determined. In addition, because of their amino acid composition, some proteins are inherently difficult to detect. Finally, different mass spectrometers, search engines, and protein assembly pipelines detect different subsets of proteins from the same biological samples. Examination of multiple biological samples, use of different detection and quantitation pipelines, and comparison between species may therefore be required to obtain the most thorough coverage of the proteome of a given cellular or subcellular fraction.

We aim to determine the core hair-bundle proteome, those proteins that are found in all bundles. Knowledge of the proteins of the bundle and their concentrations will assist in describing how the bundle is built and how it functions. Bundles are highly specialized, and specific paralogs of proteins are often selectively expressed in bundles. In other cases, there may be species-to-species variation in the identity of the best-expressed paralog. Complicating protein identification, mass spectrometry suffers from the well-known peptide assignment problem^9^, where identical peptides found in two different proteins cannot be definitively assigned to one or the other. For these reasons, it is essential to compare bundle proteomes of one species with those of other species, which should lead to the most reliable results.

We report here four separate hair-bundle proteome datasets from utricles, a vestibular organ; two are from chick and one each are from rat and mouse. We also report four matching whole-utricle datasets, one for each bundle dataset. All eight datasets, summarized in Table 1, were acquired using linear-ion-trap mass spectrometers and the proteins within them were quantified using MS2 intensities. We have previously generated chick and mouse hair bundle and utricle datasets using MS1 peak areas for quantitation^2,10^, and we show here that the ion-trap data compares favourably to the Orbitrap-acquired MS1 data. These eight ion-trap datasets, in conjunction with the four Orbitrap datasets, will be valuable for defining the key proteins of the vestibular hair bundle. To further assist in achieving this goal, we also provide combined tables with common protein grouping for the six chick datasets and, separately, for all twelve datasets analysed here.

## Methods

### Experimental design

**Figure 1:**
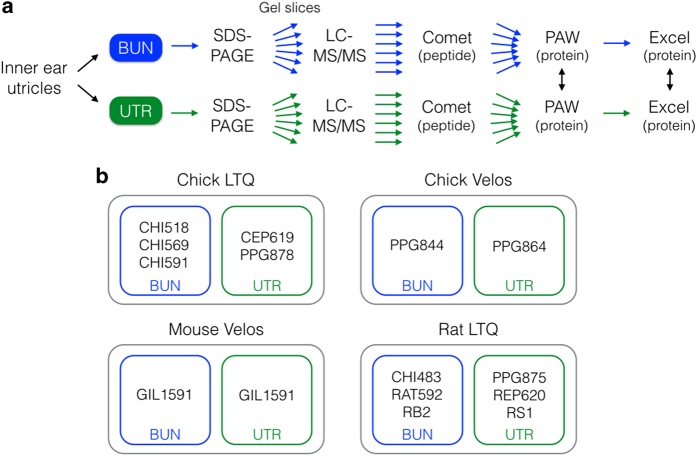
Workflow and samples. (**a**) Sample and data processing workflow for mass spectrometry analysis. (**b**) Sample groups. Each designation refers to a distinct project consisting of multiple samples, which was analysed together with identical conditions. Similar samples from different projects were pooled together in some cases.

We collected utricle hair-bundle and whole organ samples for eight separate datasets (Table 1). The structure of the utricle and the relationship of bundles to epithelium has been recently described for chick and mouse^2,10^, and rat utricles and their bundles are similar. Two complete datasets (bundles and epithelium) were derived from E19-E20 chick utricles, one from P21-P25 mouse utricles, and one from P3-P7 rat utricles. Bundle samples are referred to as ‘BUN,’ although they are modestly contaminated by cell bodies. Utricle samples are referred to as ‘UTR’; they also contain hair bundles, albeit <1% of the total protein^2,10^. The utricle samples for the chick samples were of peeled epithelium, where the epithelium is dissected away from stroma tissue; mouse and rat utricle epithelia were not easily peeled without enzyme treatment and were instead analysed as the whole organ. Each dataset contained multiple biological replicates for bundles (≥4) and for epithelium (≥4). Proteins were partially separated by SDS-PAGE, then digested into peptides. LC-MS/MS with Thermo LTQ and LTQ Velos ion-trap mass spectrometers was used to separate, fragment, and detect peptides from each of the four sets of samples. We used Comet^11^ to match peptides to protein database entries, then the in-house PAW pipeline^12^ to assemble peptides into proteins and to quantify those proteins using MS2 intensities. The workflow for bundle isolation, sample preparation, mass spectrometry, and data analysis is shown in Fig. 1. Information about the mass spectrometer instrument settings, database searching, and peptide-to-protein mapping (satisfying the Minimal Information About a Proteomics Experiment [MIAPE] requirements^13^) is presented in Supplementary Table S1.

### Hair-bundle isolation and sample preparation for mass spectrometry

Utricle hair bundles (abbreviated BUN) were purified from E19-20 chicks, P3-P7 rats, or P21-P25 mice using the twist-off method^2,14–17^. Proteins were separated by a brief SDS-PAGE run; the gel lane with separated proteins (about 1 cm) was sliced into five or six equal pieces, and the proteins reduced, alkylated, and digested with trypsin in-gel as described^2,17^. A single experiment’s worth of hair bundles or epithelium was analysed by multiple LC-MS/MS runs, one for each gel piece.

For analysis of whole utricles, the organ was dissected to remove all tissue outside the sensory epithelium region and the otolithic membrane was removed. As in previous analyses of chick, we peeled the sensory epithelium of chick utricles off of the basement membrane and connective tissue^2,17^. For mouse and rat, whole unpeeled utricles were analysed; those preparations thus contained additional proteins derived from extracellular matrix, stroma, and some nerve.

### Ion-trap mass spectrometry

Protein digests were separated by liquid chromatography using three similar ion-trap instrument setups (Supplementary Table S1), one with an LTQ linear ion trap and two others with LTQ Velos ion traps. We ran multiple biological replicates for each set of chick bundles (13 LTQ and 4 LTQ Velos experiments of 100 utricles each) and utricle epithelium (8 LTQ and 4 LTQ Velos experiments of 10 organs each), analysing the two datasets in parallel. We carried out LC-MS/MS with 5 biological replicates of rat utricle hair bundles (each from 80–100 utricles) and 7 biological replicates of whole rat utricle (each of 10 organs). There were 4 biological replicates for mouse utricle hair bundles (each from 100 utricles) and 4 biological replicates for whole mouse utricle (each of 10 organs).

#### LTQ

This configuration was used for chick LTQ and rat LTQ samples. Peptides were separated with an Agilent 1100 series capillary LC system (Agilent Technologies), then delivered to an LTQ linear ion trap mass spectrometer (Thermo Scientific) using electrospray ionization with an Ion Max source (Thermo Scientific) fitted with a 34 g metal needle (Thermo Scientific) and 2.7 kV source voltage. Xcalibur version 2.0 was used to control the system. Samples were applied at 20 μl/min to a trap cartridge (Michrom BioResources), then switched onto a 0.5×250 mm Zorbax SB-C18 column with 5 mm particles (Agilent Technologies) using a mobile phase containing 0.1% formic acid, 7–30% acetonitrile gradient over 200 min, and 10 μl/min flow rate. Collision-cell fragmentation employed helium at 9×10^−9^ bar with a normalized collision energy of 35; the mass analyser and electron multiplier were stock LTQ components. Data-dependent collection of MS/MS spectra used the dynamic exclusion feature of the instrument’s control software (repeat count equal to 1, exclusion list size of 50, exclusion duration of 30 s, and exclusion mass width of −1.0 to +4.0) to obtain MS/MS spectra of the three most abundant parent ions (minimum signal of 5,000) following each survey scan from m/z 350–1,800. The tune file was configured with no averaging of microscans, maximum inject times of 200 msec for both MS1 and MS2 scans, and automatic gain control targets of 3×10^4^ in MS1 mode and 1×10^4^ in MS2 mode.

#### LTQ Velos #1

This configuration was used for the chick Velos samples. Peptides were separated with an Agilent 1100 series capillary LC system (Agilent Technologies), then delivered to an LTQ Velos linear ion trap mass spectrometer (Thermo Scientific) using electrospray ionization with an Ion Max source fitted with a 34 g metal needle and 2.7 kV source voltage. Xcalibur version 2.0 was used to control the system. Samples were applied at 20 μl/min to a trap cartridge (Michrom BioResources), then switched onto a 0.5×250 mm Zorbax SB-C18 column with 5 mm particles (Agilent Technologies) using a mobile phase containing 0.1% formic acid, 7–30% acetonitrile gradient over 210 min, and 10 μl/min flow rate. Collision-cell fragmentation employed helium at 9×10^−9^ bar with a normalized collision energy of 35; the mass analyser and electron multiplier were stock LTQ Velos components. Data-dependent collection of MS/MS spectra used the dynamic exclusion (repeat count equal to 1, exclusion list size of 100, exclusion duration of 30 s, and exclusion mass width of −1.0 to +4.0) to obtain MS/MS spectra of the three most abundant parent ions (minimum signal of 10,000) following each survey scan from m/z 350–2,000. The tune file was configured with no averaging of microscans, a maximum inject time of 50 msec for MS1 scans and 100 msec for MS2 scans, and automatic gain control targets of 3×10^4^ in MS1 mode and 1×10^4^ in MS2 mode.

#### LTQ Velos #2

This configuration was used for the mouse Velos samples. Peptides were separated with a Waters nanoAcquity LC system, and then delivered to an LTQ Velos linear ion trap mass spectrometer (Thermo Scientific) using electrospray ionization with a Microm Captivespray source fitted with a 20 μm taper spray tip and 1.0 kV source voltage. Xcalibur version 2.1 was used to control the system. Samples were applied at 15 μl/min to a Symmetry C18 trap cartridge for 10 min, then switched onto a 75 μm×250 mm nanoAcquity BEH130C18 column with 1.7 μm particles (Waters) using a mobile phase containing 0.1% formic acid, 7.5–30% acetonitrile gradient over 140 min, and 300 nl/min flow rate. Collision-cell fragmentation employed helium at 9×10^−9^ bar with a normalized collision energy of 30; the mass analyser and electron multiplier were stock LTQ Velos components. Data-dependent collection of MS/MS spectra used the dynamic exclusion (repeat count equal to 1, exclusion list size of 500, exclusion duration of 30 s, and exclusion mass width of −1.0 to +1.5) to obtain MS/MS spectra of the ten most abundant parent ions (minimum signal of 5,000) following each survey scan from m/z 400–1,400. The tune file was configured with no averaging of microscans, maximum inject times of 200 msec for MS1 scans and 100 msec for MS2 scans, and automatic gain control targets of 3×10^4^ in MS1 mode and 1×10^4^ in MS2 mode.

### Peptide matching by Comet

For ion-trap data, MS2 scan peak lists were created from instrument RAW files using the MSConvert program (v 3.0.6705) from the Proteowizard toolkit^18,19^. Compressed text files were created using the command string ‘>msconvert --filter ‘msLevel 2’ --text –gzip’. The text files were parsed with in-house software and MS2-format files^20^ were created. Scans had to have minimum peak counts of 15 or greater, and absolute intensities of 100 or greater. Scans having 90% or greater total intensity associated with m/z values less than the precursor m/z value were assigned 1+ charge states. Scans failing this test were duplicated as 2+ and 3+ charge states.

Protein databases in FASTA format were downloaded from Ensembl (www.ensembl.org) for each species: mouse (release 76 with 52,998 sequences), chick (release 77 with 16,366 sequences), and rat (release 75 having 25,724 sequences). Database entries for known major hair-bundle proteins were checked and, in a few cases, replaced with correct and full length versions. Because Ensembl releases lack proper protein description strings, which are available from the Ensembl BioMart tool, the FASTA header lines were modified to include protein description strings. The protein databases used in the Comet searches included 179 common contaminant sequences and sequence-reversed decoy entries. Database preparation used utilities available at www.ProteomicAnalysisWorkbench.com. The respective FASTA databases are included in the ProteomeXchange datasets.

Database searching used version 2014.02 rev. 2 of Comet^11^. The searches were configured similarly for all datasets aside from the proper protein database path. Theoretical peptides were fully tryptic (cleavage at K, R except after P) with a minimum of 2 missed cleavages and within a mass range of 600–5,000 Da. Parent ion mass tolerances of 2.5 Da and average masses were specified. Static modifications of +57.0215 on cysteine residues were applied. Variable oxidations of methionine residues (M+15.9949) were also specified with a maximum of 3 modifications per peptide. Monoisotopic masses and mass tolerances of 1.0005 (0.4 fragment ion bin tolerance) were used for fragment ions. The ion series used in Comet scoring were b- and y-ions along with neutral loss peaks.

### Peptide matching by Andromeda

Chicken hair-bundle data acquired on an Orbitrap mass spectrometer^2^, previously reported as ProteomeXchange dataset PXD000104 (Data Citation 1), was reanalyzed using the the chicken Ensembl database described above (PXD002445; Data Citation 2). Searches were carried out using the search engine Andromeda^21^, built into MaxQuant version 1.5.1.2 software^22^. The default contaminants file associated with the MaxQuant download was edited to remove entries known to be present in hair bundles (e.g., actin) and to add additional impurities that entered the bundle-purification workflow (e.g., keratins, haemoglobins). Protein identifications were reported with an false discovery rate (FDR) of 5%.

### Protein assembly by PAW

For the data acquired on ion-trap mass spectrometers, we used a linear discriminant transformation to improve the identification sensitivity from the Comet analysis^12,23^. Comet scores were combined into linear discriminant function scores, and discriminant score histograms created separately for each peptide charge state (1+, 2+, and 3+), number of tryptic termini (0, 1, or 2), and modification state (unmodified or M+16 modified). Separate histograms were created for matches to forward sequences and for matches to reversed sequences for all peptides of at least 7 amino acids in length. The score histograms for reversed matches were used to estimate peptide false discovery rates (FDR) and set score thresholds for each peptide class that achieved the desired peptide FDR (typically 5% for bundles and 1% for utricles). All identifications passing the score thresholds were written to new SQT and MS2 files.

The entire set of confidently identified peptides from multiple, related samples for each species was collectively mapped to their respective protein databases using an in-house Python script. Any proteins identified by identical sets of peptides were grouped together as redundant proteins. Any proteins identified by a peptide set that was a formal subset of another protein’s peptide set were removed (basic parsimony principles). Any proteins that were not identified by at least two distinct fully tryptic peptides per biological sample (typically one 6-fraction gel run-in) were removed from the final list of identified proteins.

Highly homologous families of common proteins present in many samples pose challenges in quantitative shotgun proteomic experiments. Individual proteins within families may have few unique peptides (peptides mapped to only one protein), yet the majority of those peptides can be unique to the family. Thus, reliable quantitative information can be obtained for families even if individual family members cannot be quantified. Straightforward extensions of parsimony principles to define ‘nearly’ identical peptide sets and peptide sets that were ‘nearly’ formal subsets of larger peptide sets were used to identify protein families and group their proteins together. Definitions of unique and shared peptides were recomputed in the context of the proteins and protein families identified in the samples rather than for all proteins in the FASTA protein database. This post-processing step dramatically changes the quantitative information content of many peptides and improves protein quantitation. We have grouped proteins into families for the quantitative analyses presented here. More complete protein databases such as Ensembl have more peptide redundancy and are particularly problematic.

The in-house Python scripts have been described previously^12^. Complete search results are located in the ProteomeXchange datasets. Supplementary Table S1 contains full instrument settings, search settings, pipeline processing details, and peptide and protein FDR statistics (see also Table 2).

### Additional mass spectrometry data processing

In ion-trap mass spectrometry experiments, we quantified proteins using summed MS2 intensities of peptides identified by Comet^8,24^. Fragment ion intensities were extracted from MS2 files; the top 50 values were summed (or all values if there were between the minimum ion count of 15 and 50 values). For each protein, intensities from all peptide identifications were summed for the total intensity value for that protein of *I*. We measured for each protein its relative molar intensity (*i_m_*), the summed intensity for a protein divided by its molecular mass, divided by the sum of all intensity/mass values for all analysed proteins in a run:

(1)im=Imr∑i=1nImr

where *m*_r_ is the molecular mass of the protein and all *I/m*_r_ are summed from the experiment for a total protein number of *n*. Contaminants, including keratins, trypsin, and bovine serum albumin, were removed prior to analysis, as was all intensity associated with haemoglobin, a known contaminant of the hair-bundle preparation^15^.

In principle, *i_m_* for a protein should be identical to the mole fraction of that protein (*x_k_*), which is the number of molecules (*n_k_*) divided by the total molecules *n_tot_* in the sample (*x_k_*=*n_k_/n_tot_*). Using standard proteins in a complex protein background, we demonstrated that quantitation with MS2 intensities derived from LTQ or LTQ Velos ion-trap mass spectrometers was nearly as accurate and precise as quantitation of the same proteins using MS1 peak-area integration using an Orbitrap mass spectrometer^8^.

### Protein assembly and quantitation by MaxQuant

For the reanalyzed chick Orbitrap dataset (PXD002445), we used MaxQuant to assemble matched peptides into proteins, as well as to calculate iBAQ, a measure of protein abundance (Data Citation 2). The iBAQ value is obtained by dividing protein intensities by the number of theoretically observable tryptic peptides between 6 and 30 amino acids, and is on average highly correlated with protein abundance^8,25^. The iBAQ value for each protein was divided by the sum of all iBAQ values in a given sample, producing a relative iBAQ (riBAQ) value^8^.

### Annotation

Mouse and rat proteins were annotated by the corresponding gene name (edited for brevity) and gene symbol, which was used as the protein symbol (all caps, no italics). Because annotation of the chicken genome is incomplete and sometimes inaccurate, we manually examined both the chicken entry and Ensembl-identified orthologs of other species, particularly mouse and human, to determine the appropriate description and abbreviation. In all cases we aimed for identical ortholog protein descriptions and symbols across chicken, mouse, and rat entries.

### Comparison of datasets

A program was written using Mathematica (version 10) to compare sets of protein symbols (protein groups) between the different datasets. The program starts with the protein groups in the first dataset and compares them one by one with all protein groups in the second dataset. If any protein symbol from the second group’s protein group is found in the first dataset’s protein group, then the two protein groups are combined using a Union operation (which gives a sorted list of all the distinct elements that appear in any of the two protein groups). These operations proceed iteratively through the two datasets until a final combined list is generated. This new list is then compared with the third dataset and so on through all datasets. The final list is then compared against itself to detect protein symbols that may be in several groups, leading to a list which has protein groups that have at least one member protein symbol that is detected in one of the datasets compared.

The program then uses this final list to go back through the original datasets to aggregate the quantitative data for each group. If two or more original protein groups contribute to the aggregated protein group in the final list, then the quantitation measures (*i_m_* or riBAQ) are summed, as are the standard deviations. These values for BUN and UTR samples are reported in the final tables (Data Citation 7 and Data Citation 8). In each final table, we calculate the overall mean of the estimated molar abundance (*x_k_*) across all compared datasets for bundles and for utricles; we also calculate the overall enrichment (BUN/UTR) for each protein.

### Code availability

The MSConvert program (version 3.0.6705) is part of the Proteowizard package (http://proteowizard.sourceforge.net). Utilities used to prepare FASTA databases are available (www.ProteomicAnalysisWorkbench.com). Comet is available from http://comet-ms.sourceforge.net; we used version 2014.02 rev. 2. The custom Python code used by the PAW pipeline is available from the authors upon request, as is the custom Mathematica code used to compare proteins of different datasets.

## Data Records

All data and analysis files, described below, have been deposited to the ProteomeXchange repository (http://www.proteomexchange.org).

### Chick LTQ

The chick LTQ dataset (PXD002410; Data Citation 3) includes 126 RAW files, representing LC-MS/MS data from E19-E20 bundles (13 biological replicates) or utricles (8 biological replicates); each replicate was analysed in six runs, one for each gel slice. The dataset also includes ‘Chick_LTQ_FASTA.zip’, which contains FASTA files for searching (one with and one without reversed entries), and ‘Chick_LTQ_filtered_SQT.zip,’ which contains peak list and search result files for each replicate in MS2 and SQT formats, respectively, for all identified peptides. Two folders of PAW result files are included, one that uses spectral counts for quantitation (‘Chick_LTQ_results_files_counts’) and that uses intensities for quantitation (‘Chick_LTQ_results_files_intensity’). Threshold figures for protein assembly in the bundles and utricle datasets are in the folders labelled ‘Chick_LTQ_ThresholdFigures_BUN’ and ‘Chick_LTQ_ThresholdFigures_UTR.’ Finally, ‘Chick_LTQ_miscellaneous.zip’ contains ‘Chick_LTQ_comet.params,’ which lists the parameters used in the Comet searches; ‘Generic_instrument_settings.xls,’ which lists instrument and search settings; ‘Generic_read_me.doc,’ which provides general information about the location and contents of most files, and ‘Chick_LTQ_Comet_REV_final_table.xls,’ which is the final analysed table.

### Chick Velos

The chick Velos dataset (PXD002414; Data Citation 4) includes 48 RAW files, representing LC-MS/MS data from E19-E20 bundles (4 biological replicates) or utricles (4 biological replicates); each replicate was analysed in six runs, one for each gel slice. The dataset also includes ‘Chick_Velos_FASTA.zip’, which contains FASTA files for searching (one with and one without reversed entries), and ‘Chick_Velos_filtered_SQT.zip,’ which contains peak list and search result files for each replicate in MS2 and SQT formats, respectively, for all identified peptides. Two folders of PAW result files are included, one that uses spectral counts for quantitation (‘Chick_Velos_results_files_counts’) and that uses intensities for quantitation (‘Chick_Velos_results_files_intensity’). Threshold figures for protein assembly in the bundles and utricle datasets are in the folders labelled ‘Chick_Velos_ThresholdFigures_BUN’ and ‘Chick_Velos_ThresholdFigures_UTR.’ Finally, ‘Chick_Velos_miscellaneous.zip’ contains ‘Chick_Velos_comet.params,’ which lists the parameters used in the Comet searches; ‘Generic_instrument_settings.xls,’ which lists instrument and search settings; ‘Generic_read_me.doc,’ which provides general information about the location and contents of most files, and ‘Chick_Velos_Comet_REV_final_table.xls,’ which is the final analysed table.

### Rat LTQ

The rat LTQ dataset (PXD002416; Data Citation 5) includes 66 RAW files, representing LC-MS/MS data from P21-P25 bundles (5 biological replicates) or utricles (7 biological replicates); each replicate was analysed in five or six runs, one for each gel slice. The dataset also includes ‘Rat_LTQ_FASTA.zip’, which contains FASTA files for searching (one with and one without reversed entries), and ‘Rat_LTQ_filtered_SQT.zip,’ which contains peak list and search result files for each replicate in MS2 and SQT formats, respectively, for all identified peptides. Two folders of PAW result files are included, one that uses spectral counts for quantitation (‘Rat_LTQ_results_files_counts’) and that uses intensities for quantitation (‘Rat_LTQ_results_files_intensity’). Threshold figures for protein assembly in the bundles and utricle datasets are in the folders labelled ‘Rat_LTQ_ThresholdFigures_BUN’ and ‘Rat_LTQ_ThresholdFigures_UTR.’ Finally, ‘Rat_LTQ_miscellaneous.zip’ contains ‘Rat_LTQ_comet.params,’ which lists the parameters used in the Comet searches; ‘Generic_instrument_settings.xls,’ which lists instrument and search settings; ‘Generic_read_me.doc,’ which provides general information about the location and contents of most files, and ‘Rat_LTQ_Comet_REV_final_table.xls,’ which is the final analysed table.

### Mouse Velos

The mouse Velos dataset (PXD002415; Data Citation 6) includes 48 RAW files, representing LC-MS/MS data from P21-P25 bundles (4 biological replicates) or utricles (4 biological replicates); each replicate was analysed in six runs, one for each gel slice. The dataset also includes ‘Mouse_Velos_FASTA.zip’, which contains FASTA files for searching (one with and one without reversed entries), and ‘Mouse_Velos_filtered_SQT.zip,’ which contains peak list and search result files for each replicate in MS2 and SQT formats, respectively, for all identified peptides. Two folders of PAW result files are included, one that uses spectral counts for quantitation (‘Mouse_Velos_results_files_counts’) and that uses intensities for quantitation (‘Mouse_Velos_results_files_intensity’). Threshold figures for protein assembly in the bundles and utricle datasets are in the folders labelled ‘Mouse_Velos_ThresholdFigures_BUN’ and ‘Mouse_Velos_ThresholdFigures_UTR.’ Finally, ‘Mouse_Velos_miscellaneous.zip’ contains ‘Mouse_Velos_comet.params,’ which lists the parameters used in the Comet searches; ‘Generic_instrument_settings.xls,’ which lists instrument and search settings; ‘Generic_read_me.doc,’ which provides general information about the location and contents of most files, and ‘Mouse_Velos_Comet_REV_final_table.xls,’ which is the final analysed table.

## Technical Validation

### Comparison of biological replicates for each condition

**Figure 2:**
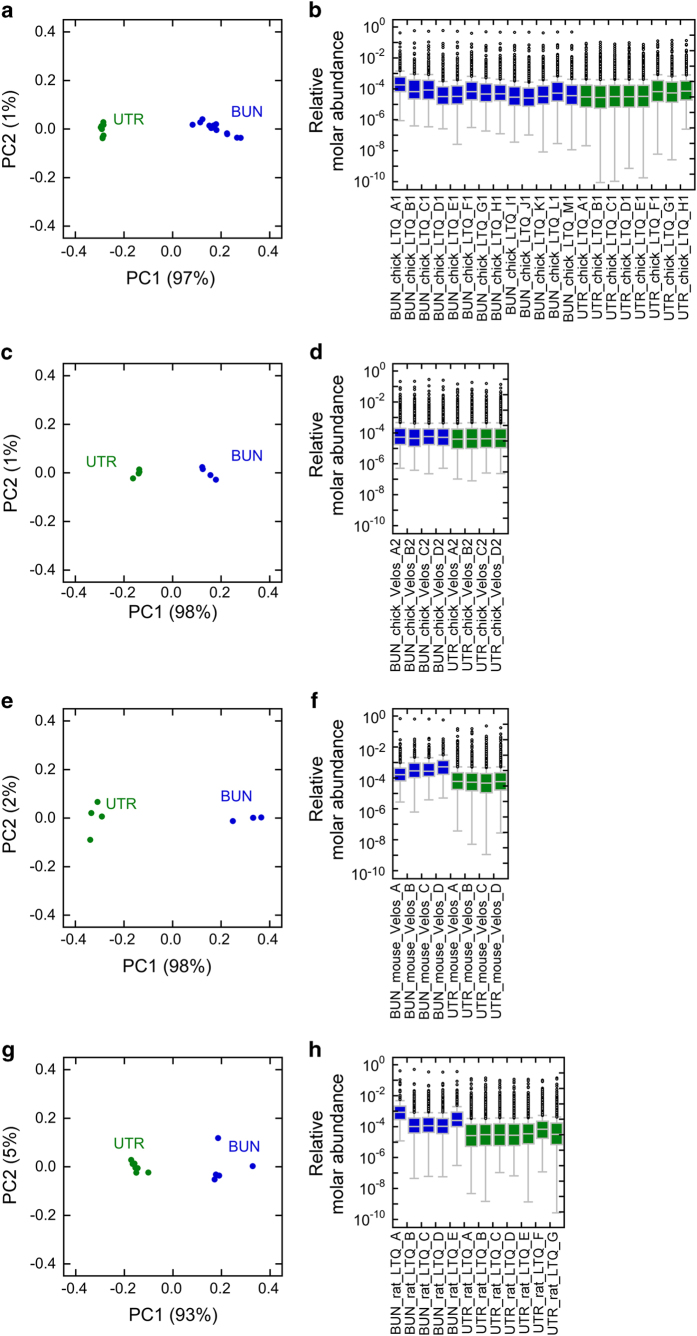
Comparison of samples making up each dataset. For each, on the left (**a**,**c**,**e**,**g**) is the principal component analysis, plotting PC1 (93–98% of variance, as indicated) against PC2 (1–5% of variance); on the right (**b**,**d**,**f**,**h**) are box plots showing relative molar abundance (im) values for all proteins in the indicated samples. (**a**,**b**) Chick LTQ. (**c**,**d**) Chick Velos. (**e**, **f**) Rat LTQ. (**g**,**h**) Mouse Velos.

Multiple biological replicates were used for bundles and utricles in each of the four datasets. Principal component analysis showed that for each dataset, the bundle and utricle samples were clearly distinguished from each other (Fig. 2), with >90% of the variance present in PC1. Box plots showing the distribution of riBAQ values for each of the samples are also shown (Fig. 2). Together the results in Fig. 2 show that the replicates for each experimental condition were sufficiently similar to each other that they could be combined.

### Comparison between datasets acquired with different mass spectrometers

**Figure 3:**
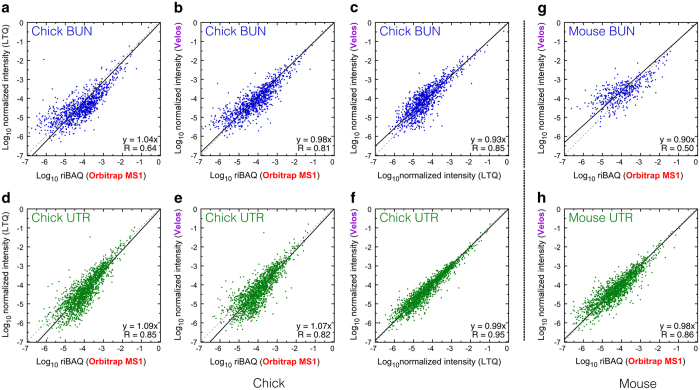
Comparison of relative abundance of proteins and protein groups between datasets. (**a-f**) Comparison of chick hair bundles (top) or utricular epithelium (bottom). Datasets are indicated in axis labels, and the fit equation and correlation coefficients are displayed. (**g**,**h**) Comparison of mouse hair bundles (**g**) and whole utricle (**h**).

We compared these ion-trap data to similar data obtained using MS1 peak intensities from an Orbitrap mass spectrometer. MS1 intensities are generally thought to be superior for quantitation compared to methods that use MS2 features, such as counts and intensities^26^. Nevertheless, using standard proteins diluted into an *E. coli* extract, we found empirically that protein abundances determined from MS1 intensities were at best only somewhat more accurate than abundances derived from MS2 intensities^8^. Regardless, we found generally good agreement between protein abundance for bundles and utricle or utricular epithelium samples determined by either Orbitrap MS1 quantitation or ion-trap MS2 intensity quantitation (Fig. 3). For chick data, the slope of the relationship between a protein’s abundance with the two mass spectrometers was ~1, although the relatively low R values (0.6–0.9) indicates that there is considerable protein-to-protein variation (Fig. 3a–f).

The mouse bundle data obtained from the Velos ion-trap mass spectrometer matched relatively poorly with similar data analysed using Orbitrap MS1 quantitation, however (Fig. 3g,h). This poor concordance may be due to the considerably smaller amounts of mouse bundles than chick bundles, as the mouse whole utricle data matched well between Orbitrap MS1 and ion trap MS2 quantitation (Fig. 3g).

### Contamination

As described previously^2^, we assessed the degree of contamination of each hair-bundle sample by measuring the enrichment of histones in the bundles. Because all histones derive from the nucleus, their relative molar abundance reflects contamination. Table 2 indicates the contamination level for histones in bundle samples from each of the datasets. The chick LTQ and LTQ Velos datasets have substantially less nuclear contamination than the chick Orbitrap dataset, indicating that the samples used for the ion-trap experiments are of high quality. These three datasets should be suitable for determination of an accurate chick bundle proteome. On the other hand, the very high level of contamination in the rat hair bundles makes them less suitable for accurate estimation of the presence and abundance of bundle proteins.

### Comparison of datasets

To assist in identifying the core proteome of vestibular hair bundles, we determined the proteins common to the four datasets described here as well as the chick Orbitrap datasets in PXD002445 and mouse Orbitrap datasets in PXD002167. We carried out two comparisons, both of combined BUN+UTR datasets: first, we compared the three chick datasets (output reported in Data Citation 7), which allowed us to confidently determine the chick bundle proteome and make FDR comparisons; second, we compared all six datasets, which allowed us to determine the core bundle proteome (output reported in Data Citation 8).

**Figure 4:**
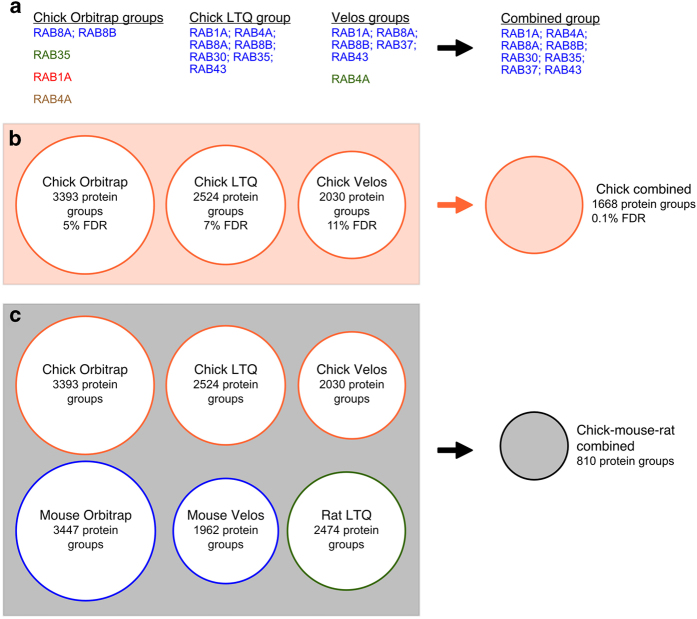
Comparison of datasets. (**a**) Example of protein groups for RAB proteins in individual datasets (left) and in the final combined protein group (right). (**b**) Chick protein group and FDR comparison. The combined group has an only somewhat lower total number of protein groups and a very low FDR. (**c**) Chick-mouse-rat protein group comparison. Comparison across the six datasets yielded a substantially smaller final set of protein groups, due both to inconsistency across the three species and to the consolidation of many protein groups.

Comparisons between datasets are complicated; depending on stochastic identification of peptides, protein grouping differs between datasets. Because we ensured that protein symbols used here matched paralogs across all three species and were appropriate for the particular accession number, we made comparisons between datasets at the level of the protein symbols. We sought to identify groups of proteins that were shared across species, and so we wrote a program that compared the datasets and generated master protein groups that included all intersecting protein groups from individual datasets. For example, if any one symbol from one protein group in dataset A was found in a protein group in dataset B, then all symbols from the two protein groups were combined. An example of this comparison for a single final protein group is shown in Fig. 4a. This process was repeated iteratively with all of the datasets. Because we sought proteins represented in all datasets, the comparison process was sequential and did not need to be performed with all dataset combinations.

Because they used the same FASTA database with identical decoy entries, we were able to carry out an FDR calculation on the common chick dataset (Fig. 4). Although we used individual datasets with relatively high FDR levels (5–11%), the common chick dataset had an FDR of only 0.1%, suggesting that the confidence in the final dataset should be relatively high. The reduction in the total number of protein groups was modest (Fig. 4b); this reduction stemmed largely from consolidation of groups but also was caused by proteins represented in only one or two datasets.

Comparison of chick, mouse, and rat datasets led to a much smaller number of final groups (Fig. 4c); this reduced number largely derived from the stringent requirement that the proteins of a group be seen in all six datasets. The resulting groups in the combined list (Data Citation 8) thus has the proteins most likely to be part of the core proteome, particularly those with enrichment over the apparent contamination level (~16%; Data Citation 8). The combined chick-mouse-rat dataset does not have an FDR calculation associated with it; reversed sequences that make up the decoy databases for chick, mouse, and rat databases are necessarily associated with different symbols (we use the ‘REV_’ appended to the accession number of the corresponding forward sequence). Nevertheless, the dramatic reduction in FDR seen when comparing the three chick datasets suggests that the chick-mouse-rat dataset has a similarly low FDR.

## Usage Notes

### Data processing with Comet

#### (1) Peak lists

Peak lists should be prepared from the RAW instrument file using MSConvert in MS2 format for processing by Comet.

#### (2) Protein databases

FASTA protein databases should be prepared with concatenated sequence-reversed decoys.

#### (3) Comet searches

Comet searches with optimum settings for the instrument type should be performed to generate SQT files for PAW processing.

### Data processing with PAW

#### (4) Peptide spectrum match (PSM) filtering

Histograms of discriminant score distributions for top hit matches to the target half of the database and for top-hit matches to the decoy half should be generated for all peptide classes (different charge states and modification states). Decoy matches relative to target matches are used to estimate PSM FDR and set thresholds. PSMs with scores exceeding thresholds are written to new MS2 and SQT files.

#### (5) Peptide to protein mapping

Peptide sequences from filtered PSMs are mapped to the proteins in the FASTA database and basic parsimony principles are applied to provide a list of confidently identified proteins meeting identification criteria. Reports using spectral counts or intensity-weighted spectral counts are generated.

#### (6) Protein grouping

Parsimony principles are extended to determine if proteins having significant peptides in common should be grouped into homologous families. After grouping, shared and unique peptide status are updated and protein quantitative measures recomputed. Additional protein and peptide reports are generated.

### Data processing with excel

The output from the PAW file was imported into Excel. The following steps were carried out:

#### (7) Trim file

First, entries labelled ‘Contaminant’ were deleted. Next, the following columns were deleted: ProtGroup, Counter, Link, Filter, Coverage, SeqLength, CountsTot, UniqTot, and UniqFrac. All ‘Total’ and ‘Unique’ columns were deleted, leaving only the ‘Corrected’ data columns for each sample. Additional contaminant entries from a user-defined list were deleted, the protein descriptions were replaced with user-described descriptions, and a protein symbol column was added.

#### (8) Calculate *i_m_*

To generate a relative abundance for each protein in its sample, we divided a protein’s total summed intensity *I* by its molecular mass *m_r_*, then divided that value by the sum of all non-contaminant *I*/*m_r_* values for that sample, yielding a relative molar abundance (*i_m_*). On average, *i_m_* is an accurate measure of protein abundance^8^.

#### (9) Calculate mean and standard deviation for biological replicates

We calculated mean and standard deviations on *i_m_* values, not on their logarithmic transformations.

#### (10) Calculate BUN/UTR enrichment

These enrichment values allow for sorting of proteins that are likely to be true bundle components from those that are contaminants.

### Comparison of datasets with Mathematica

#### (11) Prepare CSV files

Prepare a comma-separated file (.csv) for each dataset; the program looks for protein symbols in column 4, hair-bundle estimated *x_r_* (*i_m_* or riBAQ) and standard deviations in columns 6 and 7, and utricle estimated *x_r_* (*i_m_* or riBAQ) and standard deviations in columns 16 and 17.

#### (12) Run ‘Compare datasets.nb’ program

Change the base directory in the first module to the appropriate directory. Add in the file names for the datasets to be compared in the second module; also adjust ‘ntotal’ (the number of datasets compared) to the appropriate value. Run the program; ‘Combined data.csv’ and ‘Individual run summary.csv’ are the output files. We used the former file for the dataset comparisons reported in Data Citation 7 and Data Citation 8; the latter output file is useful for comparing the individual proteins groups that contributed to a given final protein group (for each dataset). We used Excel to carry out the final calculations of mean BUN and UTR *x_k_*, as well as BUN/UTR enrichment.

## Additional Information

**How to cite this article:** Wilmarth, P. A. *et al.* Hair-bundle proteomes of avian and mammalian inner-ear utricles. *Sci. Data* 2:150074 doi: 10.1038/sdata.2015.74 (2015).

## Supplementary Material



Supplementary Table S1

## Figures and Tables

**Table 1:** Samples analysed for mass spectrometry.

**Dataset name**	**Sample name**	**Developmental age range**	**Tissue source**	**Biological replicates**	**Gel slices per replicate**
Chick_LTQ	Chick_LTQ_BUN	E19-E20	Chick utricle hair bundles	13	6
Chick_LTQ	Chick_LTQ_UTR	E19-E20	Peeled chick utricle sensory epithelium	8	6
Chick_Velos	Chick_Velos_BUN	E19-E20	Chick utricle hair bundles	4	6
Chick_Velos	Chick_Velos_UTR	E19-E20	Peeled chick utricle sensory epithelium	4	6
Rat_LTQ	Rat_LTQ_BUN	P3-P7	Rat utricle hair bundles	5	5–6
Rat_LTQ	Rat_LTQ_UTR	P3-P7	Rat utricles	7	5–6
Mouse_Velos	Mouse_Velos_BUN	P21-P25	Mouse utricle hair bundles	4	6
Mouse_Velos	Mouse_Velos_UTR	P21-P25	Mouse utricles	4	6

**Table 2:** Properties of final datasets

**Dataset name**	**Sample name**	**Histone contamination**	**Post-PAW false-discovery rate**	**Post-grouping false-discovery rate**
Chick_LTQ	Chick_LTQ_BUN	3±1%	2.8%	9.4%
Chick_LTQ	Chick_LTQ_UTR	N/A	5.7%	0.9%
Chick_Velos	Chick_Velos_BUN	6±2%	2.3%	11.3%
Chick_Velos	Chick_Velos_UTR	N/A	0.6%	5.2%
Chick_Orbitrap	Chick_Orbitrap_BUN	18±12%	N/A	4.6%
Chick_Orbitrap	Chick_Orbitrap_UTR	N/A	N/A	4.6%
Rat_LTQ	Rat_LTQ_BUN	35±14%	2.6%	2.8%
Rat_LTQ	Rat_LTQ_UTR	N/A	1.0%	2.5%
Mouse_Velos	Mouse_Velos_BUN	5±7%	15%	1.3%
Mouse_Velos	Mouse_Velos_UTR	N/A	2.0%	1.1%
Mouse_Orbitrap_P23	Mouse_Orbitrap_P23_BUN	16±11%	N/A	2.6%
Mouse_Orbitrap_P23	Mouse_Orbitrap_P23_UTR	N/A	N/A	1.1%
The table includes the already described Orbitrap datasets^2,10^, as well as the ion-trap datasets that are the subject of this report. N/A, not applicable.				
